# Inactivation of chronic wasting disease prions using sodium hypochlorite

**DOI:** 10.1371/journal.pone.0223659

**Published:** 2019-10-04

**Authors:** Katie Williams, Andrew G. Hughson, Bruce Chesebro, Brent Race

**Affiliations:** Laboratory of Persistent Viral Diseases, Rocky Mountain Laboratories, National Institute of Allergy and Infectious Diseases, National Institutes of Health, Hamilton, Montana, United States of America; University of Verona, ITALY

## Abstract

Chronic wasting disease (CWD) is a fatal prion disease that can infect deer, elk and moose. CWD has now been detected in 26 states of the USA, 3 Canadian provinces, South Korea, Norway, Sweden and Finland. CWD continues to spread from endemic areas, and new foci of infections are frequently detected. As increasing numbers of cervids become infected, the likelihood for human exposure increases. To date, no cases of CWD infection in humans have been confirmed, but experience with the BSE zoonosis in the United Kingdom suggests exposure to CWD should be minimized. Specifically, hunters, meat processors and others in contact with tissues from potentially CWD-infected cervids need a practical method to decontaminate knives, saws and other equipment. Prions are notoriously difficult to inactivate, and most effective methods require chemicals or sterilization processes that are either dangerous, caustic, expensive or not readily available. Although corrosive, sodium hypochlorite (bleach) is widely available and affordable and has been shown to inactivate prion agents including those that cause scrapie, bovine spongiform encephalopathy and Creutzfeldt-Jakob disease. In the current study, we confirm that bleach is an effective disinfectant for CWD prions and establish minimum times and bleach concentrations to eliminate prion seeding activity from stainless steel and infected brain homogenate solutions. We found that a five-minute treatment with a 40% dilution of household bleach was effective at inactivating CWD seeding activity from stainless-steel wires and CWD-infected brain homogenates. However, bleach was not able to inactivate CWD seeding activity from solid tissues in our studies.

## Introduction

Chronic wasting disease (CWD) is an emerging prion disease that causes a fatal wasting syndrome in deer, elk and moose. After first detection in Colorado in the late 1960’s [1], CWD has now been reported in 26 states of the USA, three Canadian provinces, South Korea, Norway and Finland. New foci of infections are reported each year. Ranges of existing endemic areas continue to expand, and CWD prevalence within endemic areas continue to increase. As the range and prevalence of CWD increase, so does the potential for human consumption/exposures to CWD prions.

Historically, inactivation of infectious prions has been primarily a concern for research laboratories and medical facilities. However, given the recent zoonotic transmission of bovine spongiform encephalopathy (BSE) to humans in the United Kingdom [2], and the current widespread occurrence of CWD in wild and captive cervid populations, concern over prion exposure to the general public has grown. While to date, no cross-species transmission of CWD transmission to humans has been confirmed, it remains prudent to avoid consuming CWD-positive cervids and to handle CWD-infected tissues with caution. Prions, including CWD have a strong propensity to bind to surfaces including stainless steel. Hunters, meat processors, taxidermists, biologists and others that handle cervid tissues will have equipment potentially contaminated with CWD prions. Identifying an affordable, easy and effective method to inactivate CWD prions is essential for decontaminating knives, saws, and other equipment potentially in contact with CWD-infected tissues.

Prions are difficult to inactivate and numerous methods including irradiation, heat, autoclaving, chemicals, and enzymes have been tested, most with mixed success [3]. Of the effective and attainable disinfectants, sodium hypochlorite (bleach) has been shown to be effective against many prion agents including scrapie [4–8], BSE [9] and CJD [4, 10, 11]. These studies tested bleach concentrations from 2% to 40% (approx. 1,000–20,000 ppm Cl) and immersion times from 15 minutes to 24 hours. Most of the studies showed that bleach decreased prion infectivity (measured by animal bioassay) below the limit of detection and those that did not eliminate all detectable infectivity still eliminated 4–7 logs compared to no treatment. Unfortunately, none of the above published studies tested the ability of bleach to inactivate CWD prions. Since each prion strain may have different sensitivity to inactivation [12–15], it is important to confirm that CWD prions can be inactivated with bleach. One previous study did show that hypochlorous acid (HOCl) the conjugate acid of hypochlorite, which likely works through a similar oxidative mechanism as bleach to disinfect prions, was able to inactivate CWD from brain homogenates [8]. This same study also found that 40% bleach inactivated hamster scrapie seeding activity rapidly (30 seconds) from steel wires [8]. Evidence that HOCl can inactivate CWD and that bleach has the potential to work quickly motivated us to design experiments using bleach and short treatment times against CWD prions.

In our experiments we attempted to define the lowest concentrations of bleach and the minimum exposure times required to inactivate CWD. To measure the levels of prions inactivated, we used amyloid seeding activity detected by RT-QuIC as a surrogate for animal bioassay to detect prion infectivity. Several recent studies have shown that RT-QuIC seeding activity strongly correlates to bona fide infectivity and a valid surrogate for animal bioassay [8, 16–20]. First, we tested bleach against CWD-infected brain homogenate solutions to quantify levels of prion inactivation. Second, we tried to mimic two scenarios that could occur in the field and/or in game processing. Stainless steel wires were coated with CWD prions and dried to simulate how prions may adhere to knives, saws and other equipment. We also tested small pieces of whole CWD-infected tissues to see how effective bleach may be against residual bits of tissues remaining on contaminated equipment. Our data showed that strong concentrations (40%) of household bleach for a minimum of 5 minutes is effective at inactivating CWD prion seeding activity from stainless steel surfaces and brain homogenates. However, tissue penetration of bleach was poor and attempts to inactivate CWD from solid pieces of brain were not successful.

## Results

### Bleach treatments of CWD-infected brain homogenates

To quantify how much CWD amyloid seeding activity could be eliminated by our short-term bleach treatments we tested bleach decontamination of CWD-brain homogenates in parallel with mock, water treated samples. A 10μl aliquot of 10% brain homogenate from white-tailed deer (WTD-1) was mixed with 90μl of several different concentrations of bleach or water for either 1 or 5 minutes (Table 1). All bleach concentrations tested are based on undiluted Purebright brand bleach considered as 100%, which contains 6% sodium hypochlorite. Immediately following the treatment, samples were diluted 100-fold into 0.1% SDS/PBS/N2 (RT-QuIC diluent). This step was necessary to reduce any residual activity of the bleach and decrease the bleach concentration to levels previously shown to not directly inhibit the RT-QuIC assay. Due to these dilution steps, the most concentrated samples tested by RT-QuIC contained a 10^−4^ dilution of CWD-infected brain homogenate.

**Table 1:** CWD-solution decontamination.

Solution	% of household bleach added (9:1)^1^	Chlorine (ppm) in final treatment solution	Treatment time (m)	RT-QuIC^2^
WTD-1^3^ 10^−4^	none	na	na	4/4
WTD-1 10^−5^	none	na	na	4/4
WTD-1 10^−6^	none	na	na	4/4
WTD-1 10^−7^	none	na	na	4/4
WTD-1 10^−8^	none	na	na	2/4
WTD-1 CWD 10^−4^	1	450	1	8/8
	10	4,500	1	0/8
	20	9,000	1	0/8
	40	18,000	1	0/8
	1	450	5	0/8
	10	4,500	5	0/8
	20	9,000	5	0/8
	40	18,000	5	0/8
	NBH 10^−4^	none	na	5	0/4
10		4,500	5	0/4
40		18,000	5	0/4

^1^ A 10% CWD-infected brain homogenate (WTD-1) was mixed with 9 volumes of the indicated percentage of household bleach. Prior to RT-QuIC analysis treated samples were diluted another 100-fold to reduce the bleach concentration enough to avoid inhibition of the assay.

^2^ The number of RT-QuIC positive wells (see methods for criteria) is shown over the total number of wells tested.

^3^ WTD-1: CWD-positive brain homogenate pool made from 7 white-tailed deer brains

ppm, parts per million; na, not applicable; NBH, normal deer brain homogenate

**Fig 1:**
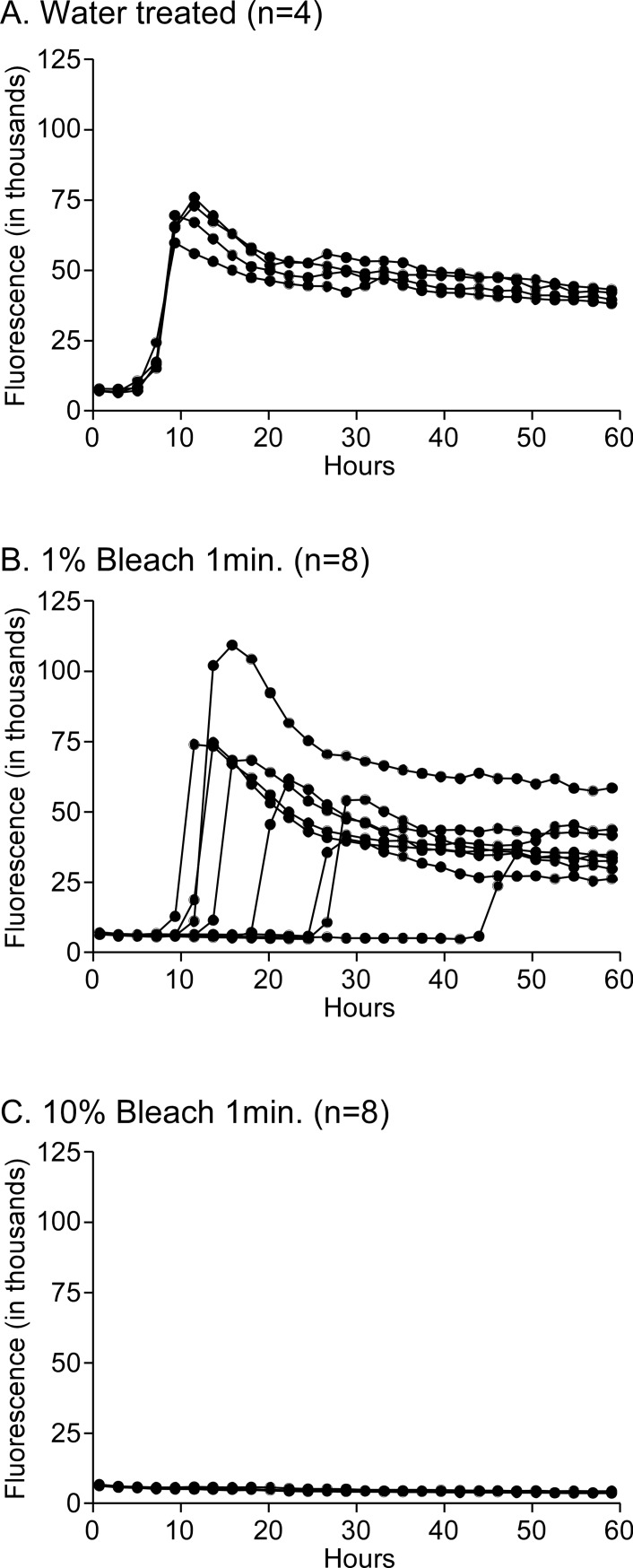
RT-QuIC analysis of water or bleach treated CWD-positive brain homogenates. A 10% brain homogenate of WTD-1 was mixed with 9 volumes of either water, 1%, or 10% bleach for 1 minute. Following treatment, the samples were diluted in RT-QuIC buffer to a 10^−4^ brain homogenate dilution and tested by RT-QuIC. Individual well data are shown for 4 replicate wells for the water treatment (A), and 8 replicate wells each for the 1% bleach treatment (B) and 10% bleach treatment (C). Note that the 1% bleach treated samples shown in B have longer lag times compared to the water treatment group in A, suggesting some inactivation occurred.

To measure the amyloid seeding activity present in water treated samples we performed serial 10-fold dilutions in RT-QuIC diluent and subjected these samples to RT-QuIC analysis. Dilutions 10^−4^ through 10^−7^ gave 100% positive reactions but 10^−8^ had only 2/4 wells react suggesting we were nearing the end-point (Table 1). Only a weak bleach solution (1%) for the shortest amount of time (1 m) was not effective against CWD in solution. Using these conditions, 8/8 wells tested positive, although their lag times were increased, suggesting partial inactivation may have occurred (compare Fig 1A to 1B). All bleach treated samples that used a concentration of 10% or higher of bleach eliminated all the amyloid seeding activity, even with only 1-minute treatments (Table 1 and Fig 1C). By comparing the untreated sample to the bleach treated samples we can see that at least 3.5 log_10_ of CWD seeding activity was removed (Table 1). This reduction is equivalent to a >3000-fold reduction in prion seeding activity.

### CWD adheres to stainless steel

**Fig 2:**
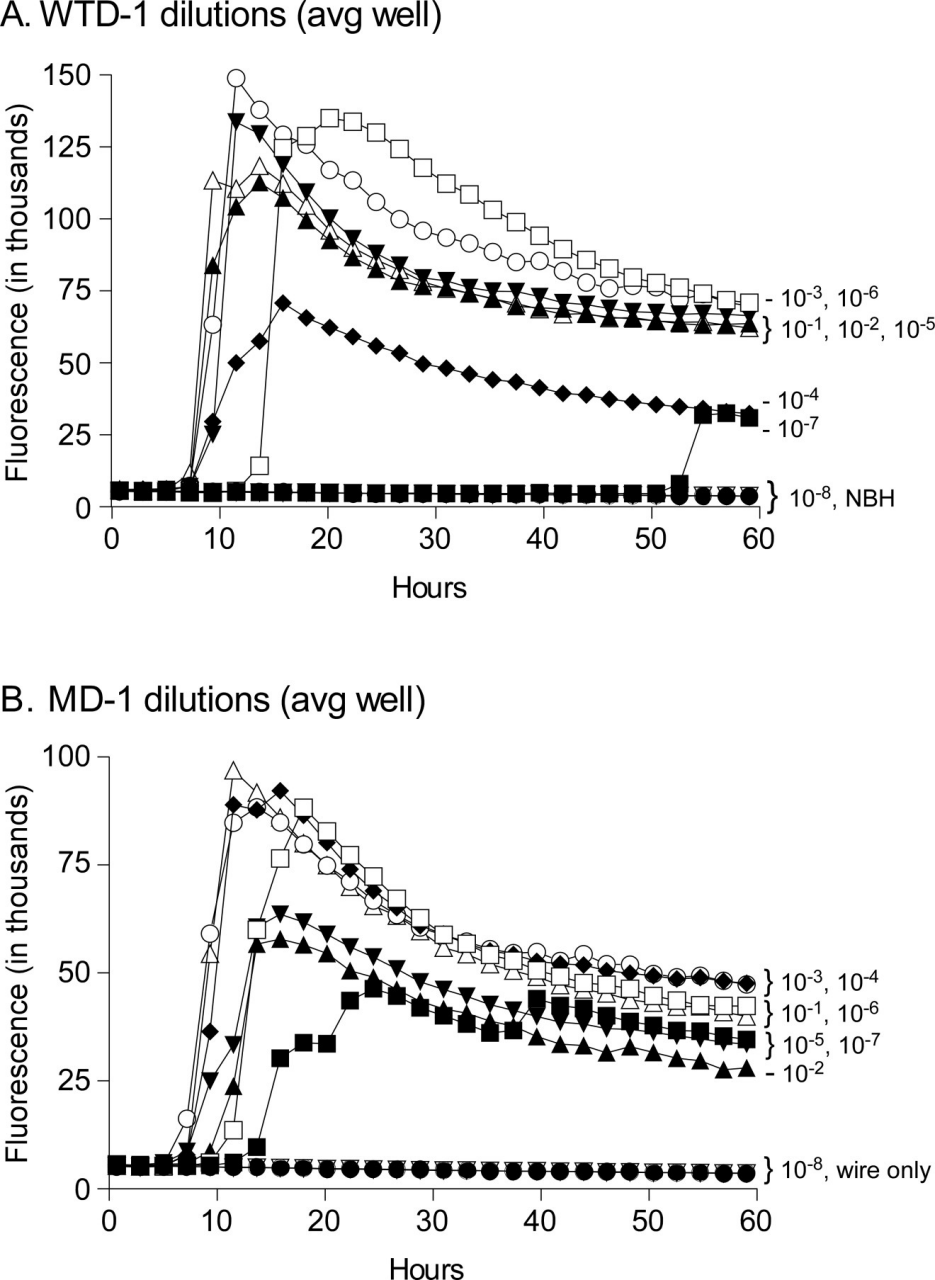
RT-QuIC analysis of stainless-steel wires exposed to varying concentrations of WTD-1 (A) or MD-1 CWD-positive brain homogenates (B). Wires were immersed for 1 hour in CWD brain homogenate dilutions from 10^−1^ to 10^−8^. Following immersion wires were washed, dried, and later placed directly into RT-QuIC reaction wells. As negative controls, wires were immersed in normal deer brain homogenate (NBH) at 10^−2^ and shown in panel A and untreated wires are shown in panel B. Each curve shown is the average fluorescence of 4 wells tested per dilution. The dilutions are shown with different symbols, and labeled to the right of each curve.

To confirm that CWD prions bound to stainless-steel wires and could be used as a decontamination model, and to identify appropriate CWD brain homogenate coating concentrations, we exposed short lengths (3-4mm) of wires to serial 10-fold dilutions of two different CWD-infected brain homogenates, WTD-1 and mule deer (MD-1). Following CWD exposure, wires were briefly rinsed and allowed to dry before being added individually to the RT-QuIC plate wells for analysis of amyloid seeding activity. All wires exposed to dilutions 10^−1^ through 10^−6^ of WTD-1 CWD (Fig 2A) and MD-1 CWD (Fig 2B) dilutions 10^−1^ to 10^−7^ were positive. Wires exposed to more dilute CWD, normal deer brain homogenates (NBH) or nothing (uncoated) were below the limit of detection by RT-QuIC assay. Based on these results we decided to expose wires to 10^−2^ and 10^−4^ dilutions of CWD brain homogenates for use in our decontamination experiments.

### Bleach treatments of CWD-contaminated wires

Wires coated with either 10^−2^ or 10^−4^ dilutions of CWD BH or NBH were dried and then exposed to various concentrations (500 ppm Cl, 1%; 5000 ppm Cl, 10%; or 20,000 ppm Cl, 40%) of freshly diluted household bleach or water for either 1 minute (Table 2) or 5 minutes (Table 3). Following treatment, the wires were washed with water to remove residual bleach, dried and stored prior to RT-QuIC analysis (see methods). RT-QuIC was performed on 8 wires (1 wire/reaction well) for each bleach concentration and treatment time. Treatments of 1 minute showed a partial decrease in seeding activity, with higher concentrations of bleach usually having better inactivation of seeding activity. While 10^−2^ wires retained some level of positivity after treatment with 40% bleach for one minute, 10^−4^ wires had no detectable seeding activity after this treatment (Table 2). Thus, lower concentrations of CWD bound to wires were easier to inactivate.

**Table 2:** One-minute bleach treatments of CWD-coated wires.

Wire coating^1^	Bleach concentration (%)	Chlorine (ppm) in treatment solution	RT-QuIC^2^
MD-1 CWD 10^−2^	untreated	na	4/4
	1	500	6/8
	10	5,000	0/8
	40	20,000	2/8
WTD-1 CWD 10^−2^	untreated	na	4/4
	1	500	4/8
	10	5,000	5/8
	40	20,000	2/8
WTD-1 CWD 10^−4^	untreated	na	4/4
	1	500	3/8
	10	5,000	2/8
	40	20,000	0/8
NBH 10^−2^	1	500	0/12
	untreated	na	0/8
NBH 10^−4^	untreated	na	0/4
No wire into rxn	none	na	0/24

^1^ The brain homogenate source and concentration used to coat the wires is listed: MD-1: CWD-positive brain homogenate pool made from 6 wild Wyoming mule deer; WTD-1: CWD-positive brain homogenate pool made from 7 white-tailed deer brains. NBH: normal deer brain homogenate, na: not applicable

^2^ The number of RT-QuIC positive wells (see methods for criteria) is shown over the total number of wells tested.

ppm, parts per million; na, not applicable; NBH, normal deer brain homogenate

**Table 3:** Five-minute bleach treatments of CWD-coated wires.

Wire coating^1^	Bleach concentration (%)	Chlorine (ppm) in treatment solution	RT-QuIC^2^
MD-1 CWD 10^−2^	untreated	Na	4/4
	0 (water only)	0	4/4
	1	500	7/8
	10	5,000	0/8
	40	20,000	0/8
WTD-1 CWD 10^−2^	untreated	na	4/4
	0 (water only)	0	4/4
	1	500	6/8
	10	5,000	2/8
	40	20,000	0/8
WTD-1 CWD 10^−4^	untreated	na	4/4
	0 (water only)	0	4/4
	1	500	1/8
	10	5,000	0/8
	40	20,000	0/8

^1^ The brain homogenate source and concentration used to coat the wires is listed: MD-1: CWD-positive brain homogenate pool made from 6 wild Wyoming mule deer; WTD-1: CWD-positive brain homogenate pool made from 7 white-tailed deer brains.

^2^ The number of RT-QuIC positive wells (see methods for criteria) is shown over the total number of wells tested.

ppm, parts per million; na, not applicable

**Fig 3:**
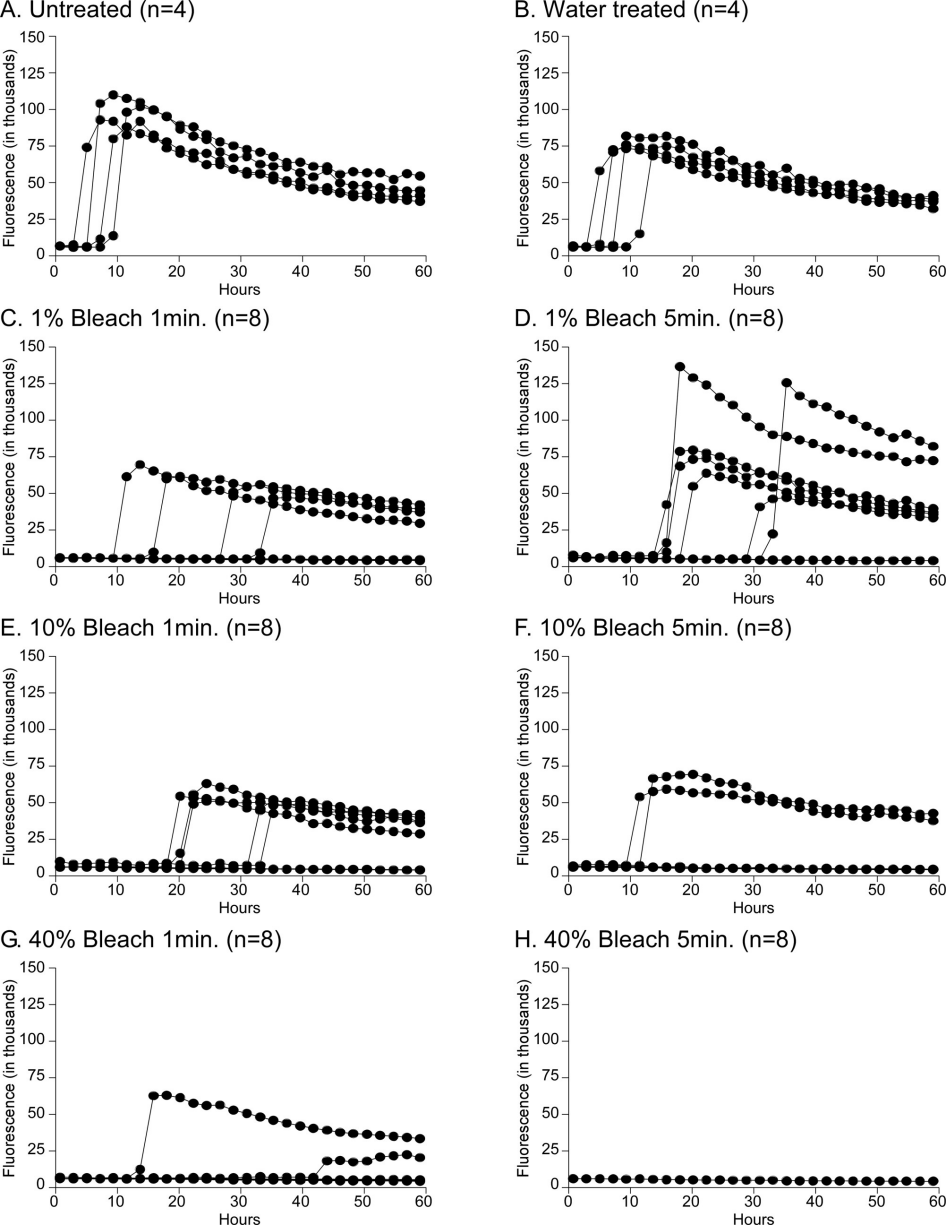
RT-QuIC analysis for treatments of WTD-1 coated wires. All wires were exposed to 10^−2^ WTD-1 CWD-positive brain homogenate, washed and allowed to dry before treatment. Wires were then immersed in either water or bleach treatments, briefly rinsed and dried before being placed into RT-QuIC plate wells for analysis of seeding activity. Bleach concentrations were tested at 1% (panels C, D), 10% (panels E, F) and 40% (panels G, H) for either 1 minute (panels C, E, G) or 5 minutes (panels D, F, H). Untreated WTD-1 coated wires are shown in panel A as a positive control. Water treated wires are shown in panel B. Individual well data are shown, 4 wells were tested for untreated (panel A) and water treated wires (panel B), 8 wires were tested for each bleach condition (panels C-H).

The only condition tested that successfully eliminated all seeding activity from wires coated with 10^−2^ dilutions of both WTD-1 or MD-1 CWD was a 5-minute treatment with 40% bleach (Table 3). Less concentrated bleach resulted in only a portion of the wells with positive seeding activity. Bleach-treated wires that retained positive seeding activity usually had increases in the lag times required for seeding activity compared to untreated positive controls (Fig 3). The combination of partially RT-QuIC positive groups and increased lag times compared to untreated controls strongly suggest that most of the CWD prions were inactivated (Fig 3). Wires immersed in water alone were not different than untreated wires, suggesting that prions adhered to wires were not simply washed off by immersion (Table 3 and Fig 3B). The overall trend from the wire decontamination experiments showed that stronger bleach concentrations and longer treatment times resulted in fewer wires having positive RT-QuIC results.

### Bleach treatments of CWD-infected brain (solid tissue)

To test the ability of bleach to inactivate CWD in solid tissues we used brains from CWD-infected or uninfected cervidized mice [21]. A 3.5 mm biopsy instrument was used to create two paired samples from similar regions on either side of mid-line for each brain. For each sample, the biopsy instrument was held vertical and downward pressure was used to cut a cylinder of tissue through the brain, centered over the thalamus area. The average weight of the biopsy samples was 37.4 mg. One sample from each pair was immersed in 700 μl water and the other in 700 μl of the specified bleach concentration. Following a specified treatment time each sample was homogenized and further processed for RT-QuIC testing. Serial ten-fold dilutions were performed to enable comparison of seeding dose 50 (SD_50_) values between treated and untreated samples. We initially tested brains from two CWD-infected mice and one uninfected mouse using 40% bleach for 5 minutes. The results from these experiments showed almost no elimination of prion seeding activity (Table 4). We then increased the treatment time to 30 minutes and tested 40% and 100% bleach treatments. Again, the results were disappointing and showed less than a 10-fold decrease in CWD-seeding activity (Table 4). Clearly, bleach is not able to inactivate prions effectively from small brain pieces under the conditions tested here.

**Table 4:** Bleach treatment of CWD-infected transgenic mouse brain.

Mouse #	Treatment, time	Dilution of brain tested by RT-QuIC^1^						Log_10_SD_50_	^2^Decrease in SD_50_
		10^−5^	10^−6^	10^−7^	10^−8^	10^−9^	10^−10^		
J399	H_2_0, 5 min	na	4/4	7/8	2/4	0/4	na	7.875	0.625
	40% Bleach, 5 min	4/4	4/4	3/4	0/4	na	na	7.25	
DA088	H_2_0, 5 min	na	4/4	0/4	0/4	0/4	na	6.5	0.25
	40% Bleach, 5 min	4/4	3/4	0/4	0/4	na	na	6.25	
J440	H_2_0, 30 min	na	8/8	8/8	7/8	4/8	1/4	9.125	0.375
	40% Bleach, 30 min	na	8/8	8/8	8/8	1/4	0/4	8.75	
J395	H_2_0, 30 min	na	8/8	8/8	6/8	3/8	1/4	8.875	0.75
	100% Bleach, 30 min	na	8/8	8/8	5/8	0/4	na	8.125	

^1^ The number of positive wells over the total number of wells tested is shown.

^2^ The calculated reduction in the seeding dose 50 (SD_50_) is shown in log_10_ following bleach treatment. In the two cases where a true end-point was not reached, we assumed that the next 10-fold dilution would have given 0 positive wells to calculate the SD_50_.

na, not applicable

## Materials and methods

### Ethics statement

The solid transgenic mouse brain tissues were obtained from archived samples produced at Rocky Mountain Laboratories (RML) under the Animal Study Proposal #2005–043. RML is an AAALAC-accredited facility and follows guidelines provided by the Guide for the Care and Use of Laboratory Animals (Institute for Laboratory Animal Research Council). CWD-infected deer brains were obtained from the Colorado Department of Wildlife and the University of Wyoming and described in greater detail below. Normal deer brains were obtained from legal hunter harvest in an area of southwest Montana considered CWD-free and tested RT-QuIC negative for CWD.

### Normal and CWD-infected brain homogenates

Two individual normal deer brains were used as negative controls. These consisted of a 3.5-year old male white-tailed deer and a <1-year old mule deer fawn. Two CWD-positive brain homogenate pools were used for our studies. White-tailed deer-1 (WTD-1) is a pool of 7 different CWD-positive brains of captive white-tailed deer from Colorado and Wyoming. Of the seven deer, five matched the deer consensus sequence and two had asparagine in place of serine at codon 138 [22]. Previous characterization of this pool demonstrated a strong PrPres immunoblot and a high level of infectivity as measure by transgenic mouse bioassay (4 x 10^8^ LD_50_ / gram) [23]. Mule deer-1 consists of 6 CWD-positive brains from free-ranging Wyoming mule deer [21]. MD-1 had a stronger PrPres immunoblot signal and slightly higher level of infectivity (5 x 10^8^ LD_50_ / gram) compared to WTD-1[23].

### Decontamination of CWD-brain homogenate

Purebright brand bleach containing 6% sodium hypochlorite was used in all of our decontamination experiments. We considered undiluted purebright bleach to be 100% bleach and the bleach dilutions tested here are based on this value, not the percentage of sodium hypochlorite. For the brain homogenate studies a 10 μl volume of 10% brain homogenate derived from a single pool of CWD infected deer (WTD-1) was mixed with 90 μl of several different concentrations of diluted bleach (1%, 10%, 20%, 40%) or water for either 1 or 5 minutes. The final chlorine ppms were 450, 4,500, 9,000 and 18,000 respectively. Immediately following the treatment, samples were diluted 100-fold into RT-QuIC diluent by performing two serial ten-fold dilutions. The first dilution was performed by adding 900 μl of RT-QuIC diluent directly to the treatment mixture. The second dilution was performed by adding 20 μl of the diluted treatment mixture into 180 μl additional RT-QuIC diluent. These steps were intended to reduce residual activity of the bleach and decrease the concentration to levels that would not inhibit the RT-QuIC assay. Samples were loaded directly into the RT-QuIC assay following the dilution steps.

### Wire preparation

Batches of sterile stainless-steel wire suture (Havel, size 000) were cut with scissors into 3–4 mm lengths. Wires were placed into wells of a 24-well tissue culture plate (15–25 wires per well) and completely immersed in 500 μl of various concentrations (see below) of either normal or CWD-infected brain homogenate pools for 1 hour with gentle agitation. Two normal deer brain homogenates and two CWD-positive brain homogenate pools (WTD-1 and MD-1) were used. After 1 hour the brain homogenates were removed by pipette and then two, 500 μl water washes were completed within the well. Following the final wash, the wires were air dried within the well for 5–10 minutes and then transferred to 1.5 mL screw cap tubes for storage prior to RT-QuIC testing or exposure to bleach. Initially, 10-fold brain homogenate dilutions from 10^−1^ to 10^−9^ were used to coat the wires with CWD. For the bleach decontamination experiments wires were exposed to either 10^−2^ or 10^−4^ dilutions of brain homogenates. Normal deer brains were obtained from a mule deer fawn (2005) and a 3.5-year-old male white-tailed deer (2018) both from an area of SW Montana believed to be CWD-free. Normal brain homogenates from these deer were negative for prion seeding activity by RT-QuIC.

### Decontamination of CWD coated wires

Wires coated with either WTD-1, MD-1 or normal deer brain homogenate were exposed to 500 μl various concentrations (500 ppm Cl, 1%; 5000 ppm Cl, 10%; or 20,000 ppm Cl, 40%) of freshly diluted household bleach (Purebright) for either 1 or 5 minutes. Directly following bleach decontamination, the bleach solution was removed by pipette followed by two 500 μl water washes. Wires were again dried and stored as described above prior to RT-QuIC analysis. Untreated controls were performed in parallel using water alone or left untreated.

### Decontamination of CWD-infected brain (solid tissue)

Whole brains from 4 clinically sick, CWD-infected or 2 uninfected cervidized mice [21] were thawed and placed on a dissection pad dorsal surface up. One CWD-infected mouse used for the 40% bleach, 5-minute exposure conditions was originally inoculated with Elk-1 CWD [23]. The remaining 3 CWD-infected mice were infected with WTD-1 CWD [23]. Days post inoculation ranged from 264–416. Using a 3.5 mm diameter biopsy instrument, two paired samples were collected from similar regions on either side of mid-line for each brain. For each sample, the biopsy instrument was held vertical and downward pressure was used to cut a cylinder of tissue through the brain, centered over the thalamus area. The average weight of the 3.5 mm diameter x ~5 mm long biopsy samples was 37.4 mg. For each pair of samples, one was placed in 700 μl of water and the other placed in 700 μl bleach (40% or, 100%) for 5 or 30 minutes. After the designated treatment times, the bleach was removed and a single 500 μl wash in water was performed. The brain tissue was weighed and homogenized to a 10% w/v brain homogenate in PBS using a bead beater. Aliquots of these homogenates were processed for RT-QuIC analysis. Dilutions from 10^−3^ to 10^−10^ were made in RT-QuIC diluent and tested by RT-QuIC for each CWD-infected treated and untreated pair and at 10^−3^ and 10^−6^ for the uninfected samples.

### RT-QuIC

RT-QuIC reactions were performed as previously described [24] using recombinant hamster 90–231 (Ha rPrP) (Accession No. KO2234) with some minor modification for the wire experiments.

For analysis of stainless-steel wires, single wires were transferred into wells containing 100 μl of the RT-QuIC reaction mix (10 mM phosphate buffer (pH 7.4), 300 mM NaCl, 0.1 mg/mL rPrPsen substrate, 10 μM thioflavin T (ThT), 1 mM ethylenediaminetetraacetic acid tetrasodium salt (EDTA), 0.002% SDS).

For bleach treated and untreated brain homogenates, samples were diluted in 0.1% SDS (sodium dodecyl sulfate, Sigma)/PBS/N2 (Gibco) to reduce bleach concentrations prior to RT-QuIC analysis. The most concentrated samples analyzed by RT-QuIC contained 0.4% bleach (200 ppm) and brain tissue at a 10^−4^ dilution. 2 μL sample volumes were added to reaction wells of a black 96-well, clear bottom plate (Thermo Scientific) containing 98 μL of RT-QuIC reaction mix, resulting in final concentrations of 0.002%, 10 mM phosphate buffer (pH 7.4), 300 mM NaCl, 0.1 mg/mL rPrPsen substrate, 10 μM thioflavin T (ThT), 1 mM ethylenediaminetetraacetic acid tetrasodium salt (EDTA). The plate was then sealed with a plate sealer film (Sarstedt) and incubated at 50°C in a BMG FLUOstar Omega plate reader with a repeating protocol of 1 min shaking (700 rpm double orbital) and 1 min rest throughout the indicated incubation time. ThT fluorescence measurements (450±10 nm excitation and 480±10 nm emission; plate bottom read) were taken every 45 min. For all runs the gain was set at 1600.

At least eight negative control wells (wells or wires containing NBH +/- bleach treatment) were run on each plate. In the conditions and experiments shown here, spontaneous fluorescence in negative samples were rarely noted prior to 50 h reaction time. Well data was scored from 0–50 hours. Positive control samples had maximum fluorescence values ranging from 59,000–210,000 and, negative controls had values ranging from 3,500–7,500. Based on these clear differences between baseline negative levels and positive reactions, we defined the minimum required fluorescence (threshold) for experimental samples to be considered positive at 15,000 ThT fluorescence. This value is three times higher than the average baseline (5,180) fluorescence of negative control wells and is 14 standard deviations above the average fluorescence of negative control wells. Setting the threshold at this point prevents false positives due to any baseline chatter.

## Discussion

Increased prevalence of CWD has been a growing concern for those handling cervid tissues. As the range and number of CWD infected cervids expands, so does the potential for human exposure. While no human cases of CWD have been confirmed to date, it remains prudent to handle CWD-infected tissues with caution. A practical method for decontaminating equipment contaminated with CWD prions is needed to reduce potential exposures. Since bleach is widely available and has previously been shown to inactivate other prion diseases [4–11] it was logical to directly test the ability of bleach to inactivate CWD prions. Our study tested the ability of bleach to inactivate CWD prion seeding activity in three different scenarios; CWD-positive brain homogenates, CWD prions bound to steel wires, and CWD-positive solid brain tissue. We found that both the concentration of bleach and the time of treatment are critical for inactivation of CWD prions. A 40% bleach treatment for 5 minutes successfully eliminated detectable prion seeding activity from both CWD-positive brain homogenate and stainless-steel wires bound with CWD. However, even small solid pieces of CWD-infected brain were not successfully decontaminated with the use of bleach. This was not surprising as bleach has traditionally been used as a surface disinfectant since it has poor tissue penetration and much lower efficacy when excessive organic matter is present [25]. Therefore, it is very important to thoroughly clean and remove solid pieces of tissue prior to surface decontamination with bleach. Methods to dispose of solid CWD-contaminated tissues and materials should follow local governmental guidelines.

Traditionally, the efficacy of prion decontamination methods has been measured using animal bioassay [3, 12]. However other methods such as the cell-based assay [26] and the RT-QuIC have recently been utilized to assess decontamination methods [8, 27]. The use of the RT-QuIC as a substitute for animal bioassay is sensible based on the strong correlation between prion amyloid seeding activity and prion infectivity in rodent models [16, 17], and with CWD prions [18–20]. In addition, the RT-QuIC has numerous advantages over animal bioassay, including greater sensitivity, rapid turnaround, cost savings and a reduction in animal use.

The use of stainless-steel wires for prion decontamination studies has been used by several groups previously to mimic surgical instrument contamination [8, 12, 15, 26–33]. Often equipment used by hunters, meat processors and biologists are also made of, or coated with stainless steel, therefore we applied the stainless-steel wire model here to mimic the surfaces of knives, saws, and processing equipment contaminated with CWD prions. We were able to demonstrate that even very dilute levels of CWD will bind to wires (Fig 2) and the CWD-coated wires can be added directly into the RT-QuIC assay with no need for further dilution, eliminating any potential loss in sensitivity.

In our experiments we used CWD-infected brains, or CWD-infected brain homogenates derived from clinically ill animals that contained high levels of CWD prions as a source of the CWD prions [23]. This was intentional, to create a worst-case scenario situation for CWD prion contamination. In typical field dressing and meat processing situations, the tissues handled most frequently such as muscle, skin, and connective tissues, all have much lower levels of infectious prions than brain and spinal cord. In our experiments, it was clear that wires coated with less CWD required lower concentrations of bleach to inactivate prion seeding activity (Tables 2 & 3, compare WTD-1 10^−2^ to 10^−4^).
